# Information-Theoretic Intrinsic Plasticity for Online Unsupervised Learning in Spiking Neural Networks

**DOI:** 10.3389/fnins.2019.00031

**Published:** 2019-02-05

**Authors:** Wenrui Zhang, Peng Li

**Affiliations:** Department of Electrical and Computer Engineering, Texas A&M University, College Station, TX, United States

**Keywords:** intrinsic plasticity, spiking neural networks, unsupervised learning, liquid state machine, speech recognition, image classification

## Abstract

As a self-adaptive mechanism, intrinsic plasticity (IP) plays an essential role in maintaining homeostasis and shaping the dynamics of neural circuits. From a computational point of view, IP has the potential to enable promising non-Hebbian learning in artificial neural networks. While IP based learning has been attempted for spiking neuron models, the existing IP rules are *ad hoc* in nature, and the practical success of their application has not been demonstrated particularly toward enabling real-life learning tasks. This work aims to address the theoretical and practical limitations of the existing works by proposing a new IP rule named SpiKL-IP. SpiKL-IP is developed based on a rigorous information-theoretic approach where the target of IP tuning is to maximize the entropy of the output firing rate distribution of each spiking neuron. This goal is achieved by tuning the output firing rate distribution toward a targeted optimal exponential distribution. Operating on a proposed firing-rate transfer function, SpiKL-IP adapts the intrinsic parameters of a spiking neuron while minimizing the KL-divergence from the targeted exponential distribution to the actual output firing rate distribution. SpiKL-IP can robustly operate in an online manner under complex inputs and network settings. Simulation studies demonstrate that the application of SpiKL-IP to individual neurons in isolation or as part of a larger spiking neural network robustly produces the desired exponential distribution. The evaluation of SpiKL-IP under real-world speech and image classification tasks shows that SpiKL-IP noticeably outperforms two existing IP rules and can significantly boost recognition accuracy by up to more than 16%.

## 1. Introduction

Neural plasticity, the brain's ability to adapt in response to stimuli from the environment, has received increasing interest from both a biological and a computational perspective. As one such main self-adaptive mechanism, intrinsic plasticity (IP) plays an important role in temporal coding and maintenance of neuronal homeostasis. Behaviors of IP have been discovered in brain areas of many species, and IP has been shown to be crucial in shaping the dynamics of neural circuits (Marder et al., 1996). In particular, Baddeley et al. (1997) observed the exponentially distributed neuron responses in visual cortical neurons. Such responses may aim at allowing neurons to transmit the maximum amount of information, e.g., measured by the highest entropy, to their outputs with a constrained level of firing activity. Discovered in individual biological neurons, IP changes the excitability of neurons through modification of voltage-gated channels (Desai et al., 1999).

From a computational point of view, one of the early biological IP models was explored on the Hodgkin-Huxley type neurons where a number of voltage-gated conductances were considered (Stemmler and Koch, 1999). Since then, much IP mechanism research has been conducted for different kinds of artificial neurons. On the one hand, Triesch (2005) first proposed a mathematical approach to derive an IP rule based on the sigmoid neuron model. This work used the Kullback Leibler (KL) divergence from an exponential distribution to the actual output firing rate distribution to derive an adaptation rule for the neuron to generate responses following the exponential distribution. Based on the same principle, an IP rule for hyperbolic tangent neurons was also proposed (Schrauwen et al., 2008). On the other hand, IP can control average firing activity and aid synapses to undergo Hebbian modification via STDP depending upon their history of use (Watt and Desai, 2010). Furthermore, it was shown that an improvement in performance could be obtained when the reservoir of an echo state network (ESN) is adapted using IP such that the neurons in the network can autonomously tune themselves to the desired output distribution (Schrauwen et al., 2008).

As the third generation of artificial neural networks, it has been shown that spiking neural networks (SNN) are more computationally powerful than previous generations of neural networks (Maass, 1997). However, developing effective intrinsic plasticity (IP) mechanisms for SNNs is a challenging problem. Several empirical IP rules were proposed for SNNs, however, without a rigorous theoretical foundation. Lazar et al. (2007) presented an IP rule by which a spiking neuron's firing threshold voltage changes by a fixed value per update based on whether the neuron fired or not. However, this method cannot precisely determine when and how much the firing threshold voltage should be changed in different situations, and there is no clear understanding of the optimality of the resulting IP behavior. Li and Li (2013) presented an approach in which the parameters of the IP rule derived for sigmoid neurons in Li (2011) were empirically mapped to ones for spiking neurons. Since this rule is derived based on the sigmoid neuron model which is significantly different from the spiking neuron model, the property of this IP rule remains elusive when it is applied to adapt the output firing activity of spiking neurons. Recently, Li et al. (2018) proposed an IP rule according to the inter-spike-interval (ISI). However, similar to Lazar et al. (2007), this method only constraints the ISI into a certain range but does not have a rigorous target for adapting the output response. Moreover, Panda and Roy (2017) proposed another homeostasis mechanism called Non-Hebbian Plasticity which decays synaptic weights based on the activity of postsynaptic neurons. It can control the reservoir neurons responses to match the firing rate profile of the input and also avoid weight crowding caused by STDP. This Non-Hebbian Plasticity is based on synaptic plasticity which is different from IP, the intrinsic neuronal plasticity. As discussed in Watt and Desai (2010), both of them are homeostatic plasticity mechanisms and observed in biological neurons. They can work together for homeostatic regulation.

From an information theoretical perspective, it may hypothesize that a nervous cell maximizes the mutual information between its input and output. Neglecting the intrinsic uncertainty of the output, i.e., the output uncertainty after the input is known, the above target is equivalent to maximizing the output entropy. To this end, it is instrumental to note that the exponential distribution of the output firing rate attains the maximum entropy under the constraint of a fixed mean firing rate (Bell and Sejnowski, 1995). Thus, inspired by the IP rule for sigmoid neurons of Triesch (2005), we aim to derive an IP rule for spiking neurons while minimizing the difference between the output firing rate distribution and the targeted exponential distribution. However, there are several significant challenges in deriving such a rule. Unlike artificial neurons whose output is in the form of firing rate, spiking neurons generate responses in the form of discrete spikes. As a result, firing rate information, as well as its dependency on the input, must be appropriately characterized from discrete spike times, which has not been established before under the context of intrinsic plasticity. Besides, it is not clear how a proper expression of the entropy of the output firing rate distribution (or its difference from the targeted exponential distribution) can be derived and robustly maximized (minimized) in an online fashion.

In this article, we approach the above challenges as follows. First, we derive a differentiable transfer function bridging the input current strength and output firing rate when the input level is fixed based on the leaky integrate-and-fire(LIF) model. This transfer function is referred to as the firing-rate transfer function (FR-TF). It shall be noted that FR-TF can correlate the dynamic evolution of the output firing activity measured as averaged firing rate as a function of a received input over a sufficiently long timescale. Next, with this transfer function, we derive an information-theoretical intrinsic plasticity rule for spiking neurons, dubbed *SpiKL-IP*, to minimize the KL-divergence from the exponential distribution to the output firing rate distribution. We further present an online version of the SpiKL-IP rule for minimizing our KL-divergence based loss function in a way analogous to the stochastic gradient descent (SGD) method, which is widely adopted for training deep learning neural networks. Finally, we address two practical issues to ensure the proper operation and robustness of the proposed online IP rule. Among the two issues, it is desirable to apply the proposed IP tuning using the instantaneous input current and the measured output firing rate, allowing seamless consideration of the potentially dynamically changing current input. However, this creates a mismatch to the underlying FR-TF transfer function, which is addressed by making the online IP rule dependent only on the output firing rate such that the LIF model parameters are tuned based on the input/output activities of long timescales. Under various settings, the outputs of targeted spiking neurons converge robustly to the desirable exponential distribution under the proposed SpiKL-IP rule.

We evaluate the learning performance of the proposed IP rule for real-world classification tasks under the context of the liquid state machine (LSM). When applied to the reservoir neurons of LSM networks, our rule produces significant performance boosts. Based on the TI46 Speech Corpus (Liberman et al., 1991), the SpiKL-IP rule boosts the recognition accuracy by 6% for single-speaker English letter recognition and by up to more than 16% for the challenging task of multiple-speaker English letter recognition. For image classification using the CityScape dataset (Cordts et al., 2016), our proposed method can improve the accuracy by more than 2%.

The rest of this article is organized as follows. Section 2 first introduces previous intrinsic plasticity working on spiking neurons. Then, it presents the derivation of the proposed firing-rate transfer function (FR-TF) and the complete online IP rule. Section 3 demonstrates the application of the proposed IP under various simulation settings. Finally, section 4 concludes this work.

## 2. Materials and Methods

### 2.1. Previous IP Rules for Spiking Neurons

Unlike other types of artificial neurons, instead of producing continuous-valued firing rates, spiking neurons generate spike trains, which are not differentiable at the times of spikes. Thus, the relationship among the input, parameters of the neuron model, and the output firing rate become obscure. This is perhaps partially why intrinsic plasticity has not been deeply investigated for spiking neurons. A few empirical IP rules were proposed for spiking neuron model, which unfortunately lack rigor.

Lazar et al. (2007) proposed an IP rule to adjust the firing threshold voltage as follows

(1) $$\begin{array}{l} V_{th,i}\left(t+1\right)=V_{th,i}\left(t\right)+\eta \left(x_{i}\left(t\right)-\frac{k}{N}\right), \end{array}$$

*V*_*th,i*_ is the threshold of the neuron *i*, η the learning rate which is chosen to be small, *x*_*i*_(*t*) the sum of Dirac delta functions and it is 1 if the neuron fires an output spike at time *t* and 0 otherwise, *k* and *N* some chosen constants. This rule drives a neuron to spike on average *k* out of *N* times. It only targets setting the mean firing rate to a chosen value by adapting the firing threshold but does not attempt to generate the optimal output response, i.e., the optimal firing rate distribution.

Li (2011) derived an IP rule that tunes sigmoid neurons to follow the Weibull distribution in the same way as in Triesch (2005). Li and Li (2013) adopted this rule for spiking neurons by merely substituting the tuning parameters of the sigmoid neuron model to the parameters for spiking neurons, namely *rR* and *rC*, which are the reciprocals of the leaky resistance and membrane capacitance, respectively. As analyzed by the authors, this rule can make the firing activity of a spiking neuron at a “low but not too low” level. However, since this rule results from a simple mapping from the sigmoid neuron IP rule, it may not produce the optimal firing rate distribution for spiking neurons.

Li et al. (2018) proposed an approach based on the Izhikevich model (Izhikevich, 2003) to adjust the output firing activity such that the inter-spike-interval (ISI) is set between some limits specified by *T*_*min*_ and *T*_*max*_. This basic idea is the same as the one in Lazar et al. (2007) but using a different neuron model. Again, this rule aims at helping the neuron to generate responses at a desired firing rate level without optimizing the output distribution to maximize the information content.

As discussed above, the existing IP rules for spiking neurons are empirical in nature and are not derived with a rigorous optimization objective in mind. Furthermore, no success in real-world learning tasks has been demonstrated. We address these limitations by rigorously deriving an IP rule that robustly produces the targeted optimal exponential firing rate distribution and leads to significant performance improvements by realistic speech and image classification tasks.

### 2.2. Firing-Rate Transfer Function

The leaky integrated-and-fire (LIF) model is one of the most prevalent choices for describing dynamics of spiking neurons. This model is given by the following differential equation (Gerstner and Kistler, 2002)

(2) $$\begin{array}{l} \tau_{m}\frac{dV}{dt}=-V+Rx \end{array}$$

where *V* is the membrane potential, *x* the input current, τ_*m*_ the time constant of membrane potential with value τ_*m*_ = *RC*, where *R* and *C* are the effective leaky resistance and effective membrane capacitance. Once the membrane potential *V* exceeds the firing threshold *V*_*th*_, the neuron generates a spike, and the membrane potential is reset to the resting potential, which is 0*mV* in our case. A refractory period of duration *t*_*r*_ is also considered after a spike is generated during which *V* is maintained at 0*mV*.

Before presenting the proposed SpiKL-IP rule for spiking neurons, we shall first establish the relationship between the input current and the resulting output firing rate. This relationship is not evident since the response is in the form of spikes and it depends on the cumulative effects of all the past input. As a result, it is difficult to evaluate the output firing rate of spiking neurons at each time point under a varying input. We deal with this difficulty by deriving the proposed firing-rate transfer function (FR-TF) where the input is assumed to be constant. In other words, FR-TF correlates the dynamic evolution of the output firing activity measured as averaged firing rate as a function of a received input over a sufficiently long timescale.

Assuming that the input current *x*_0_ is constant and integrating (2) with the initial condition that *V*(*t*^(1)^) = 0 gives the interspike interval $T_{isi}=t^{\left(2\right)}-t^{\left(1\right)}$ (Gerstner and Kistler, 2002)

(3) $$\begin{array}{l} T_{isi}=t_{r}+\tau_{m}ln\frac{Rx_{0}}{Rx_{0}-V_{th}},\;Rx_{0}>V_{th}. \end{array}$$

where the constraint of *Rx*_0_ > *V*_*th*_ comes from the fact that only when the constant input current is sufficiently large, the neuron can generate spikes. Since both the input *x*_0_ and *T*_*isi*_ are constant, the mean output firing rate of the spiking neuron is given by

(4) $$\begin{array}{l} y=\frac{1}{T_{isi}}=\frac{1}{t_{r}+\tau_{m}ln\frac{Rx_{0}}{Rx_{0}-V_{th}}},\;Rx_{0}>V_{th}. \end{array}$$

In this way, we obtain the transfer function of spiking neurons under the condition that it has constant input so that this relation between input and output can be used in the deriving process. Since this function can only represent spiking neurons with a fixed input, to distinguish the spiking neurons and this transfer function, when referring to firing-rate model neurons, it means the neurons with this firing-rate transfer function (4).

**Figure 1:**
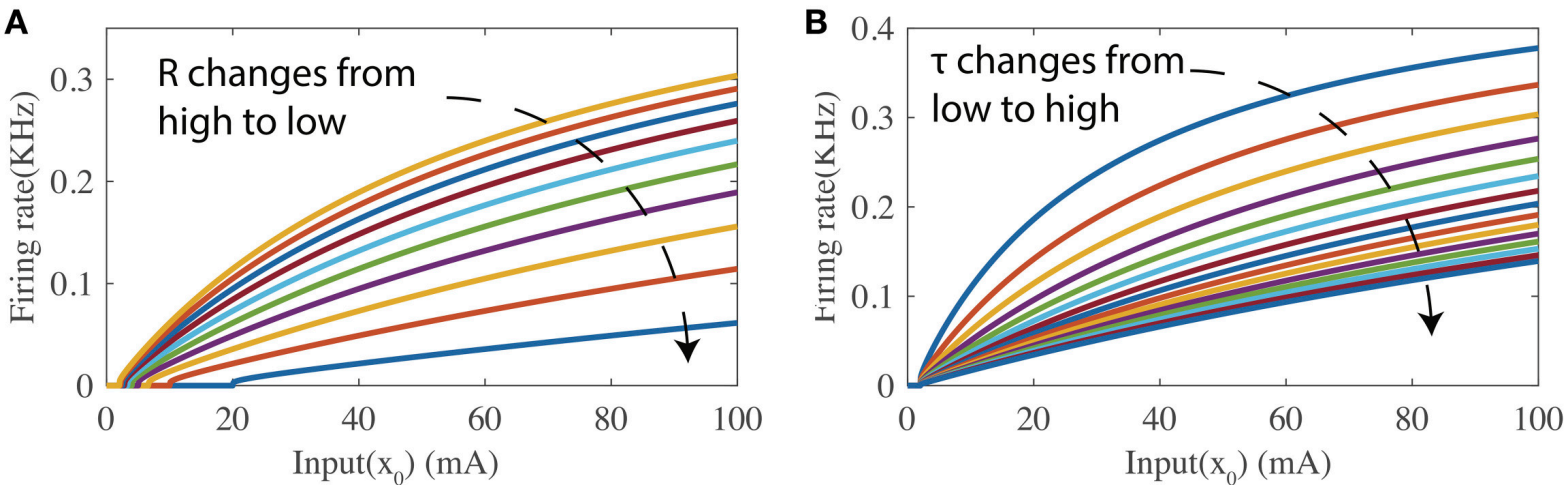
The firing-rate transfer function (FR-TF). **(A)** As a function of the leaky resistance *R*, and **(B)** as a function of the membrane time constant τ_*m*_.

Figure 1 shows two tuning curves of the firing-rate transfer function where the input current level is swept while either the leaky resistance *R* or the membrane time constant τ_*m*_ is held at a specific value. As shown in Figure 1A, changing *R* while fixing τ_*m*_ modifies both the bias and curvature of the tuning curve. Figure 1B illustrates that τ_*m*_ controls the curvature of the tuning curve when *R* is fixed. In the following part, the proposed SpiKL-IP Rule is based on tuning *R* and τ_*m*_. Note that separately adjusting *R* and τ_*m*_ requires a neuron to vary its capacitance in response to its activity while changing capacitance is not observed in biological neurons to date.

### 2.3. Proposed SpiKL-IP Rule

Based on the presented firing-rate transfer function (4), we now take an information-theoretical approach to derive the SpiKL-IP rule to minimize the KL-divergence from the exponential distribution to the output firing rate distribution. We will show how the SpiKL-IP rule can be cast into an online form to adapt *R* and τ_*m*_, and then address one practical issue to ensure the proper operation and robustness of the proposed online IP rule.

#### 2.3.1. The Basic SpiKL-IP Rule

**Figure 2:**
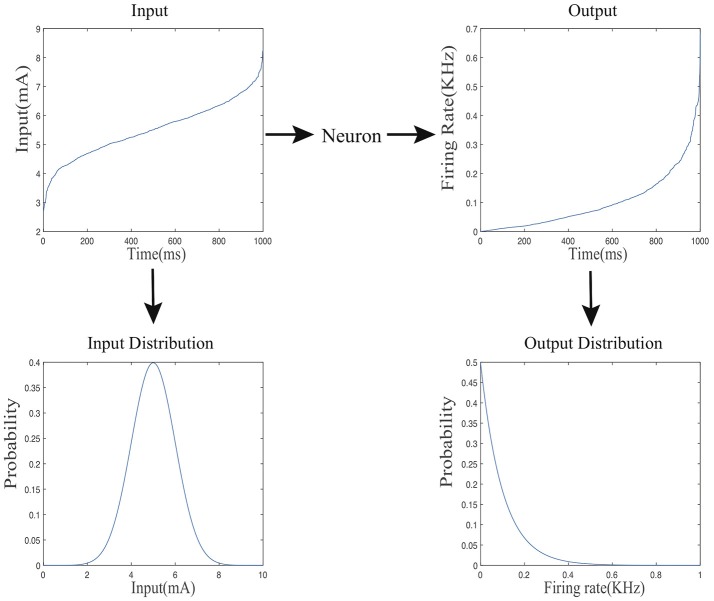
The mapping from the input current distribution to the output firing rate distributing of a neuron.

We consider the information processing of a given spiking neuron as it receives stimuli from external inputs or other neurons in the same network over a dataset, mimicking part of the lifespan of the biological counterpart. We define the input and output firing rate probability distributions for each spiking neuron in the following way. As shown in Figure 2, the input current level *X* varies across different time points, it forms an input probability distribution over the course of the entire process denoted by *f*_*x*_(*x*). Accordingly, the output firing rate *Y* varies over time and forms an output probability distribution denoted by *f*_*y*_(*y*).

The goal of the SpiKL-IP rule is to obtain an approximately exponential distribution of the output firing rate at a fixed level of metabolic costs. In a biological perspective, exponential distributions of the output firing rate have been observed in mammalian visual cortical neurons responding to natural scenes and allow the neuron to transmit the maximum amount of information given a fixed level of metabolic costs (Baddeley et al., 1997).

From an information-theoretic point of view, Bell and Sejnowski (1995) argued that a neuron might self-adapt to maximize the mutual information of the input *X* and the output *Y*, a measure for the amount of information about the input obtained from the output, or vice versa

(5) $$\begin{array}{l} I\left(Y,X\right)=H\left(Y\right)-H\left(Y|X\right), \end{array}$$

where *H*(*Y*) is the entropy of the output while *H*(*Y*|*X*) represents the amount of entropy (uncertainty) of the output which does not come from the input. Under the assumption that the output noise *N* is additive and there is no input noise, the conditional entropy can be simplified to *H*(*Y*|*X*) = *H*(*N*) (Nadal and Parga, 1994; Bell and Sejnowski, 1995) which does not depend on the neural parameters. Thus, maximizing *I*(*Y, X*) is equivalent to maximizing *H*(*Y*) (Bell and Sejnowski, 1995). To this end, it is instrumental to note when the mean of the distribution is kept constant, the exponential distribution corresponds to the largest entropy among all probability distributions of a non-negative random variable. This leads to the conclusion that the exponential distribution with a targeted mean shall be the optimal distribution for the output firing rate, where the mean specifies the practical constraint on energy expenditure. In addition, in this work, all neurons are implemented using the LIF model which is noiseless and no noise is added explicitly to the neuronal dynamics, which means that *H*(*N*) = 0 (Gerstner and Kistler, 2002). The exponential distribution is given by

(6) $$\begin{array}{l} f\left(x\right)=\mu exp\left(-\mu x\right),\;x>=0, \end{array}$$

where μ is the mean of the distribution.

Inspired by the IP rule for sigmoid neurons in Triesch (2005), we derive the SpiKL-IP rule for spiking neurons while minimizing the KL-divergence from a targeted exponential distribution to the actual output firing rate distribution, where Kullback Leibler divergence (KL-divergence) is used as a difference measure as follows

(7) $$\begin{array}{l} D=d\left(f_{y}\left(y\right)||f_{exp}\right)\\ =\int f_{y}\left(y\right)log\left(\frac{f_{y}\left(y\right)}{\frac{1}{\mu }exp\left(\frac{-y}{\mu }\right)}\right)dy\\ =\int f_{y}\left(y\right)log\left(f_{y}\left(y\right)\right)dy+\int f_{y}\left(y\right)\left(\frac{y}{\mu }\right)dy\\ +\int f_{y}\left(y\right)log\mu dy, \end{array}$$

where *y* and *f*_*y*_(*y*) denote the output, and the output firing rate distribution, respectively, and μ is the mean value of the targeted exponential distribution. The smaller the KL-divergence *D* is, the closer the exponential distribution is to the output distribution. In (7), since ∫*f*_*y*_(*y*)*dy* = 1 the third integral evaluates to a fixed value of *logμ*. Minimizing KL-Divergence *D* by adapting *R* and τ_*m*_ reduces to minimize the first two integrals, giving rise to the following loss function

(8) $$\begin{array}{l} L=\int f_{y}\left(y\right)log\left(f_{y}\left(y\right)\right)dy+\int f_{y}\left(y\right)\left(\frac{y}{\mu }\right)dy\\ =E\left[log\left(f_{y}\left(Y\right)\right)+\frac{Y}{\mu }\right]. \end{array}$$

**Figure 3:**
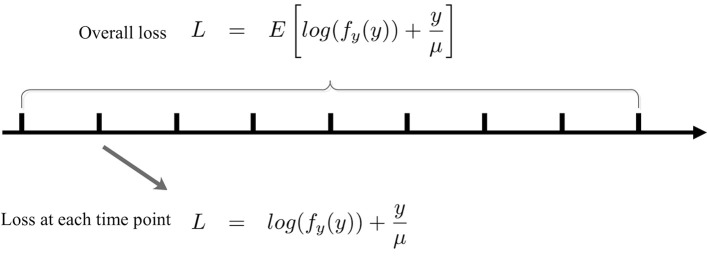
Online SpiKL-IP learning: minimization of the KL divergence at each time point during the training process.

Note that (8) is in terms of an expectation over the entire output distribution. Now, we convert (8) into an online form that is analogous to the stochastic gradient descent method with a batch size of one. To make SpiKL-IP amenable for online training, using a proper stepsize we discretize the entire training process into multiple small time intervals each in between two adjacent time points as shown in Figure 3. The input level to the spiking neuron at each time point is considered as an individual observation or training example. In this way, the adjustment of the tunable parameters is not delayed until the output firing rate distribution is collected after the entire dataset is applied to the neuron (or neural network). Instead, these parameters are adjusted as the neuron experiences a given input example at each time point in an online manner. To do this, the following loss function that corresponds to the received input example is minimized at each time point *t*

(9) $$\begin{array}{l} L\left(t\right)=log\left(f_{y}\left(y\left(t\right)\right)\right)+\frac{y\left(t\right)}{\mu }, \end{array}$$

where *y*(*t*) denotes the output firing rate *Y* observed at time *t*. From now on, we drop the explicit dependency of *y*(*t*) and *x*(*t*) (observed input level at *t*) on *t* for notational simplicity. Recognizing that the output probability distribution relates to the input counterpart by Papoulis and Pillai (2002)

(10) $$\begin{array}{l} f_{y}\left(y\right)=\frac{f_{x}\left(x\right)}{\frac{\partial y}{\partial x}} \end{array}$$

and substituting it into (9) leads to

(11) $$\begin{array}{l} L\left(t\right)=log\left(f_{x}\left(x\right)\right)-log\left(\frac{\partial y}{\partial x}\right)+\frac{y}{\mu }, \end{array}$$

which can be further simplified to

(12) $$\begin{array}{l} \hat{L}\left(t\right)=-log\left(\frac{\partial y}{\partial x}\right)+\frac{y}{\mu }, \end{array}$$

as *log*(*f*_*x*_(*x*)) is a function of the input probability distribution and does not depend on *R* and τ_*m*_.

The online SpiKL-PI rule is based upon the partial derivatives of (9) with respect to *x*, *R* and τ_*m*_. We first shall compute the derivatives of the output firing rate *y*(*t*) with respect to *x*, *R*, τ_*m*_. We make use of the firing rate transfer function (4) whose application at each time point *t* is justified if the input *x*(*t*) changes slowly with respect to the chosen stepsize and the averaged output firing rate measure is used, and obtain

(13) $$\begin{array}{l} \frac{\partial y}{\partial x}=\frac{y^{2}\tau_{m}V_{th}}{x\left(Rx-V_{th}\right)} \end{array}$$

(14) $$\begin{array}{l} \frac{\partial y}{\partial R}=\frac{y^{2}\tau_{m}V_{th}}{R\left(Rx-V_{th}\right)} \end{array}$$

(15) $$\begin{array}{l} \frac{\partial y}{\partial \tau_{m}}=\frac{t_{r}y^{2}-y}{\tau_{m}}. \end{array}$$

Taking (13) into account, the partial derivatives of the loss function (9) with respect to *R* and τ_*m*_ are found to be

(16) $$\begin{array}{l} \frac{\partial L}{\partial R}=\frac{\partial }{\partial R}\left(-log\left(\frac{\partial y}{\partial x}\right)+\frac{y}{\mu }\right)\\ =\frac{\partial }{\partial R}\left(-\left(2log\left(y\right)-log\left(Rx-V_{th}\right)\right)+\frac{y}{\mu }\right)\\ =\frac{\left(\frac{y^{2}}{\mu }-2y\right)\tau_{m}V_{th}+Rx}{R\left(Rx-V_{th}\right)} \end{array}$$

and

(17) $$\begin{array}{l} \frac{\partial L}{\partial \tau_{m}}=\frac{\partial }{\partial \tau_{m}}\left(-log\left(\frac{\partial y}{\partial x}\right)+\frac{y}{\mu }\right)\\ =\frac{\partial }{\partial \tau_{m}}\left(-\left(2log\left(y\right)+log\tau_{m}\right)+\frac{y}{\mu }\right)\\ =\frac{1+\frac{1}{\mu }\left(t_{r}y^{2}-y\right)-2t_{r}y}{\tau_{m}}, \end{array}$$

respectively, which gives the following online IP rule

(18) $$\begin{array}{l} R=R-\eta_{1}\frac{\partial L}{\partial R}\\ =R+\eta_{1}\frac{\left(2y-\frac{y^{2}}{\mu }\right)\tau_{m}V_{th}-Rx}{R\left(Rx-V_{th}\right)},\;Rx>V_{th}\\ \tau_{m}=\tau_{m}-\eta_{2}\frac{\partial L}{\partial \tau_{m}}\\ =\tau_{m}+\eta_{2}\frac{2t_{r}y-1-\frac{1}{\mu }\left(t_{r}y^{2}-y\right)}{\tau_{m}},\;Rx>V_{th}. \end{array}$$

where η_1_ and η_2_ are learning rates, μ the constant value depending on the desired mean of the output firing rate. The condition that *Rx* > *V*_*th*_ comes from the transfer] function (4).

#### 2.3.2. Practical Considerations

While (18) has the critical elements of the proposed online IP rule, its direct implementation, however, has been experimentally shown to be unsuccessful, i.e., it can neither train spiking neurons to generate output firing rates following the exponential distribution nor improve SNN learning performance for real-world classification tasks. The problem has to do with the fact that one underlying assumption behind the firing rate transfer function (FR-TF) (4) and hence the IP rule (18) is that the input current is constant or changes over a sufficiently slow timescale. However, in a practical setting, the total postsynaptic input received by a spiking neuron does vary in time, and the rate of change depends on the frequency of firing activities of its presynaptic neurons. With the internal dynamics, the output firing level of a spiking neuron cannot immediately follow the instantaneous current input, e.g., it is possible that the output firing rate is still low while the input current has increased to a high level. As a result, the assumption on the input current is somewhat constraining, and its violation leads to the ineffectiveness of IP tuning.

On the other hand, it is worth noting that the FR-TF captures the correlation between the average input current and the output firing rate over a long timescale. In the meantime, the proposed IP rule aims to adapt spiking neurons to produce a desired probability distribution of the output firing rate. In other words, the objective is not to tune each instance of the output firing rate. Instead, it is to achieve a desirable collective characteristic of the output firing rate measured by an exponential distribution. In some sense, the FR-TF correlates the input and output correspondence in a way that is meaningful for the objective of online IP tuning.

To find a solution to the above difficulty, we remove the dependency on the instantaneous input current from the IP rule of (18) by substituting the input *x* using the output firing rate *y* using the transfer function (4). More specifically, a new variable *W* is defined by *W* = *Rx*−*V*_*th*_, which can be expressed using *y* based on (4) as

(19) $$\begin{array}{l} W=\frac{V_{th}}{e^{\left(\frac{1}{\tau_{m}}\left(\frac{1}{y}-t_{r}\right)\right)}-1}. \end{array}$$

Making use of (19), (18) is converted to a form which only depends on *y*

(20) $$\begin{array}{l} R=R+\eta_{1}\frac{2y\tau_{m}V_{th}-W-V_{th}-\frac{1}{\mu }\tau_{m}V_{th}y^{2}}{RW},\;y>0.\\ \tau_{m}=\tau_{m}+\eta_{2}\frac{2t_{r}y-1-\frac{1}{\mu }\left(t_{r}y^{2}-y\right)}{\tau_{m}}. \end{array}$$

As can be seen, the rule in (20) adjusts the two parameters only based on the output firing rate *y*. Substituting the instantaneous value of *x* by the firing rate *y* based on the firing rate transfer function effectively operates the IP rule based on the averaged input/output characteristics over a longer timescale.

Note that the condition that *Rx* > *V*_*th*_ in (18) is changed to an equivalent form of *y* > 0 in (20). A closer examination of Figure 1 shows that the firing rate transfer functions are not differentiable around *y* = 0 (*Rx* = *V*_*th*_). Interpreting differently, the proposed IP tuning can operate only when the output firing rate is nonzero. To further improve the robustness of the proposed IP rule, the tuning in (20) is only activated when *y* > δ with δ being small such as 1 Hz. When *y* ≤ δ, *R* and τ_*m*_ are increased and decreased respectively to bring up the output firing activity.

Putting everything together, the final SpiKL-IP rule is

(21) $$\begin{array}{l} R=\{\begin{array}{ccc} R+\eta_{1}\frac{2y\tau_{m}V_{th}-W-V_{th}-\frac{1}{\mu }\tau_{m}V_{th}y^{2}}{RW},\; & y & >\delta \\ R+\eta_{1}\alpha_{1},\; & y & \leq \delta \end{array}\\ \tau_{m}=\{\begin{array}{ccc} \tau_{m}+\eta_{2}\frac{2t_{r}y-1-\frac{1}{\mu }(t_{r}y^{2}-y)}{\tau_{m}},\; & y & >\delta \\ \tau_{m}-\eta_{2}\alpha_{2},\; & y & \leq \delta \end{array} \end{array}$$

where α_1_ and α_2_ are chosen to be small.

**Figure 4:**
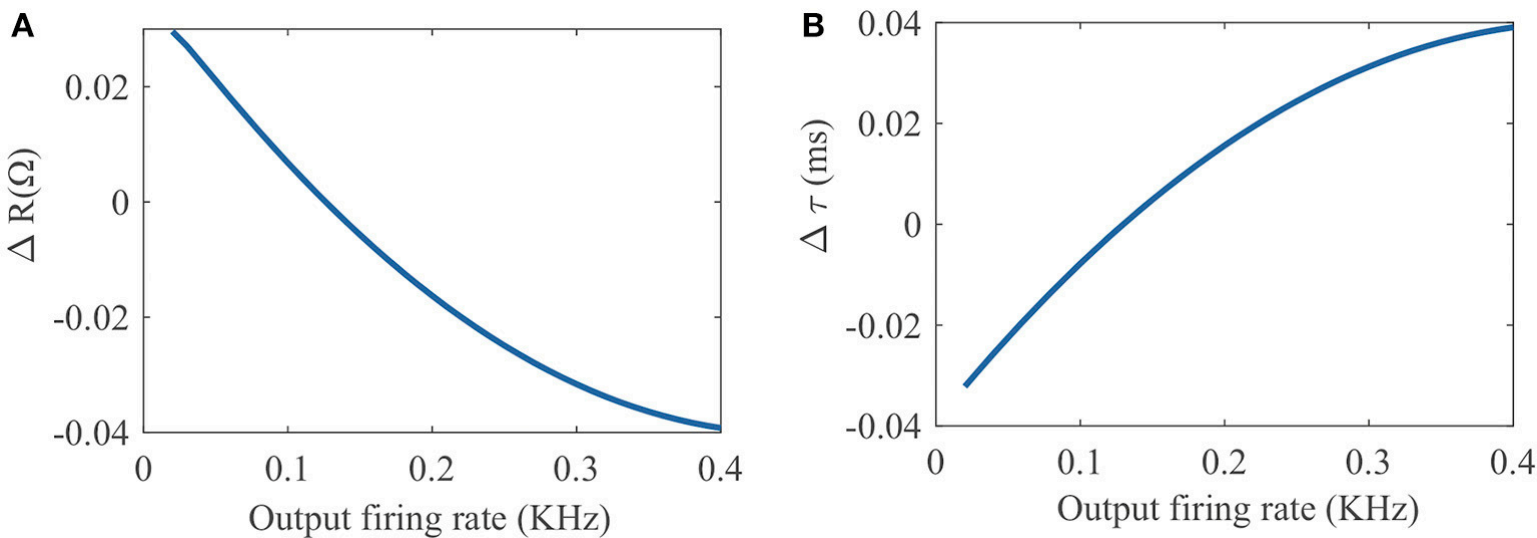
Tuning characteristics of one-time application of SpiKL-IP at different output firing rate levels starting from a chosen combination of *R* and τ_*m*_ values *R* and τ_*m*_. **(A)** Tuning of the leaky resistance *R*, and **(B)** tuning of the membrane time constant τ_*m*_.

To provide an intuitive understanding of the proposed SpiKL-IP rule, Figure 4 shows how *R* and τ_*m*_ are altered by one-time application of SpiKL-IP at different output firing rate levels starting from a chosen combination of *R* and τ_*m*_ values.

## 3. Results

To demonstrate the mechanisms and performances of the proposed SpiKL-IP rule, we conduct three types of experiments by applying SpiKL-IP to single neuron as well as a group of spiking neurons as part of a neural network. First, we show that when applied to a single neuron whose behavior is governed by the firing-rate transfer function (4) the proposed rule can tune the neuron to produce the targeted exponential distribution of the output firing rate even under a time-varying input. Then, we apply SpiKL-IP to a single spiking neuron as well as a group of spiking neurons to demonstrate that our rule can robustly produce the desired output firing distribution in all tested situations even although it is derived from the FR-TF which is based on the assumption that the input is constant. Finally, we demonstrate the significant performance boosts achieved by SpiKL-IP when applied to real-world speech and image classification tasks. Furthermore, we compare SpiKL-IP with two existing IP rules for spiking neurons (Lazar et al., 2007; Li and Li, 2013). In this article, we name the IP rule in Lazar et al. (2007) as the Voltage-Threshold IP rule and one in Li and Li (2013) as the RC IP rule.

The following simulation setups are adopted in each experiment. We simulate the continuous-time LIF model in section 2.2 using a fixed discretization time step of 1*ms* according to which all neuronal activities are evaluated in lockstep. To measure the firing rate of each spiking neuron as a continuous-valued quantity over time under a constant of varying input, we use the intracellular calcium concentration *C*_*cal*_(*t*) as a good indicator of the averaged firing activity over a chosen timescale

(22) $$\begin{array}{l} \frac{dC_{cal}\left(t\right)}{dt}=-\frac{C_{cal}\left(t\right)}{\tau_{cal}}+\underset{i}{\sum }\delta \left(t-t_{i}\right), \end{array}$$

where τ_*cal*_ is the time constant, and the output firing spikes are presented by a series of Dirac delta functions. According to (22), the calcium concentration increases by one unit when an output spike is generated and decays with a time constant τ_*cal*_ (Dayan and Abbott, 2001). The time-varying output firing rate is measured using the normalized calcium concentration

(23) $$\begin{array}{l} y\left(t\right)=\frac{C_{cal}\left(t\right)}{\tau_{cal}}. \end{array}$$

### 3.1. Single Neurons Modeled by FR-TF

We apply the proposed SpiKL-IP rule to a single neuron modeled based on the firing-rate transfer function (4). The parameters of the neuron and SpiKL-IP are set as follows: *V*_*th*_ = 20*mV*, *t*_*r*_ = 2*ms*, and μ = 0.2*KHz*. In addition, the tuning ranges for *R* and τ_*m*_ are set to [1Ω, 1024Ω] and [1*ms*, 1, 024*ms*] with *R* and τ_*m*_ initialized to 64Ω and 64*ms*, respectively. The input current level at each time point is randomly generated according to a Gaussian distribution with the mean of 7*mA* and variance of 1*mA* as well as a uniform distribution between [0.5*mA*, 5.5*mA*] in a way that is similar to the setups in Triesch (2005); Li and Li (2013). For both cases, a total of 10, 000 time points are considered.

**Figure 5:**
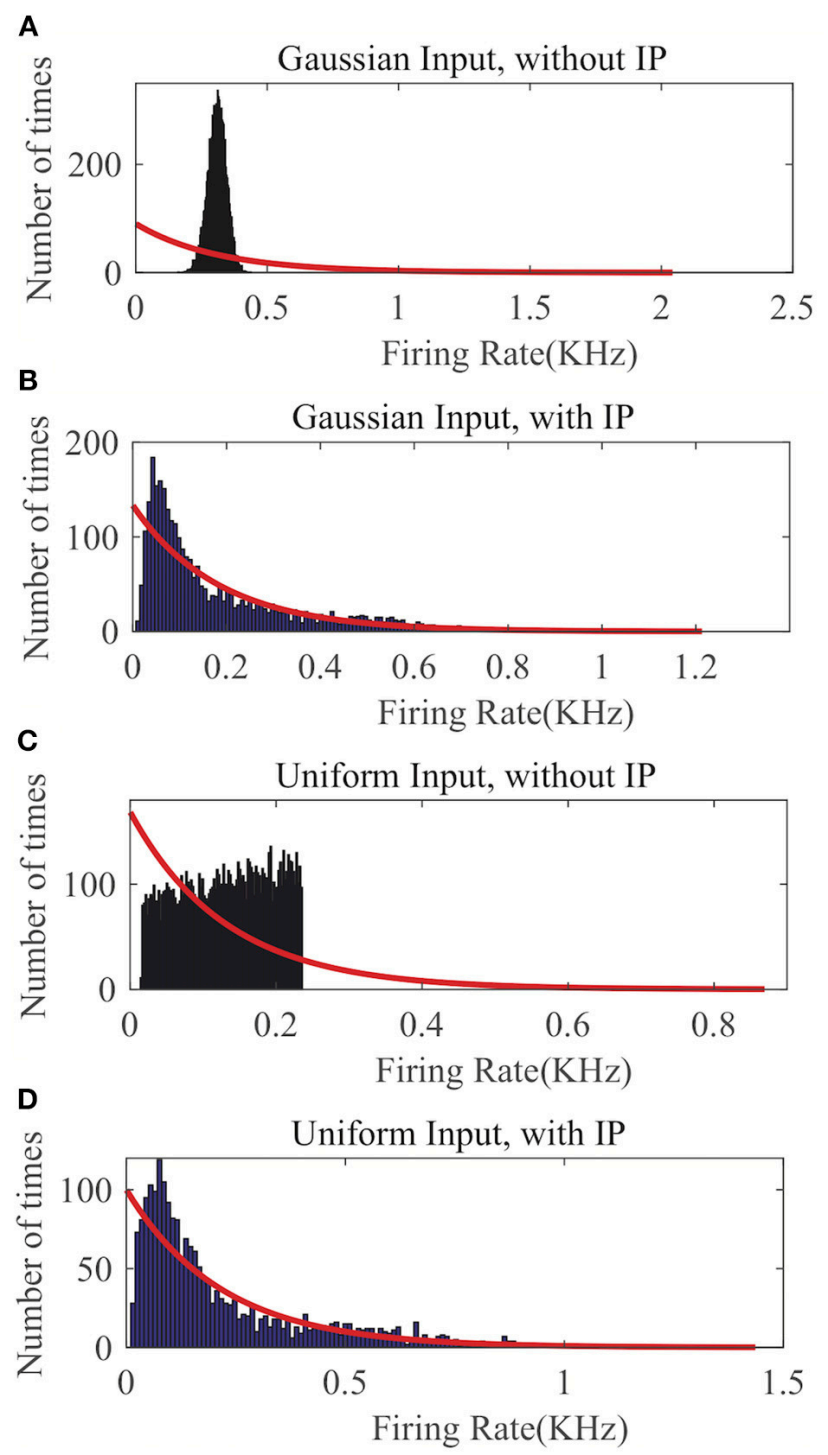
The output firing-rate distributions of a single neuron characterized using the firing-rate transfer function and driven by randomly generated current input following a Gaussian or Uniform distribution. **(A)** Gaussian input without IP tuning, **(B)** Gaussian input with the SpiKL-IP rule, **(C)** uniform input without IP tuning, and **(D)** uniform input with the SpiKL-IP rule. The red curve in each plot represents the exponential distribution that best fits the actual output firing rate data.

In Figure 5, we compare the recorded output firing rate distribution when no IP tuning is used with the one that is produced by the proposed SpiKL-IP rule under two random input distributions. In each plot of Figure 5, we fit the actual firing histogram with to a closest exponential distribution (red curve). It is evident from Figures 5A,C that without IP tuning the output firing distribution is far from the targeted optimal exponential distribution with the maximum entropy. With the application of SpiKL-IP, however, the output distribution can be trained to almost converge to the desirable exponential distribution under two dramatically different input distributions. Note that since the simulation time stepsize is 1*ms*, the output firing rate is bound by 1*KHz*. This creates a subtle difference between the actual and the exponential distribution at the tails of the two distributions, which is negligible in practice. These results indicate that the proposed IP rule can robustly maximize the information contained in the output firing rate distribution by tuning it toward the exponential distribution regardless of the input distribution.

### 3.2. Leaky Integrate-and-Fire Spiking Neurons

Since SpiKL-IP is based on the firing-rate transfer function which only characterizes the behavior of LIF neurons over a large timescale, it is interesting to test SpiKL-IP using LIF neurons. The parameters for the spiking neurons and SpiKL-IP are set as follow: *V*_*th*_ = 20*mV*, *t*_*r*_ = 2*ms*, μ = 0.2*KHz*, τ_*c*_ = 64*ms* with *R* and τ_*m*_ initialized to 64Ω and 64*ms*, respectively. The tuning ranges for *R* and τ_*m*_ are again set to [1Ω, 1, 024Ω] and [1*ms*, 1, 024*ms*], respectively.

**Figure 6:**
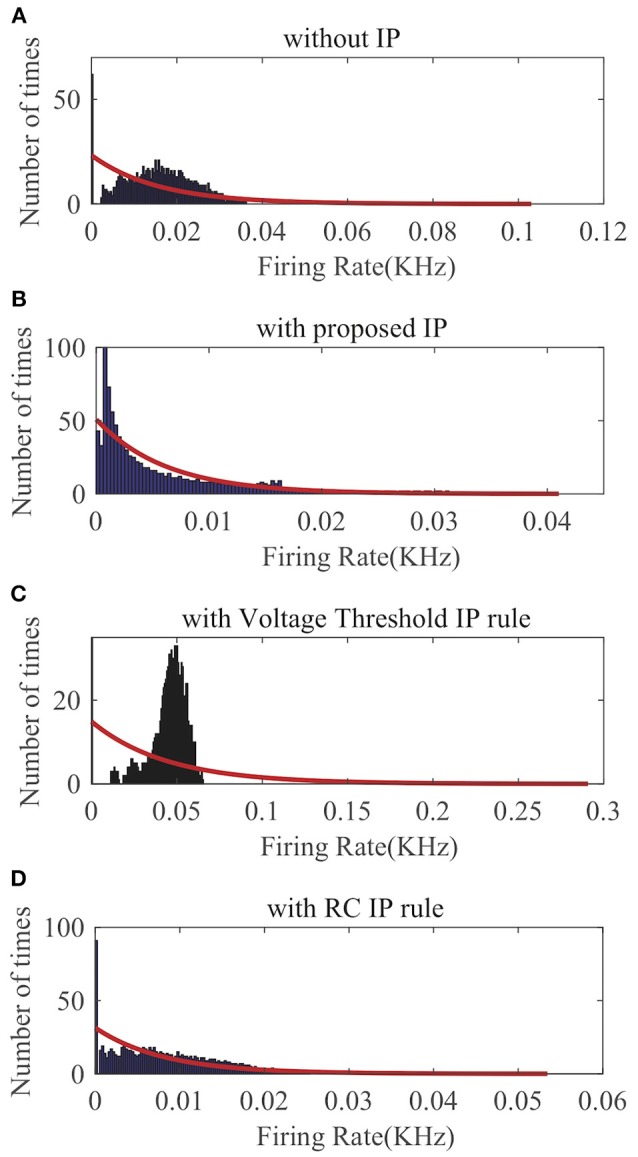
Output firing rate distributions of a single spiking neuron: **(A)** without IP tuning, **(B)** with proposed SpiKL-IP rule, **(C)** with the Voltage Threshold IP rule, and **(D)** with the RC IP rule. The red curve in each plot represents the exponential distribution that best fits the actual output firing rate data.

First, we apply SpiKL-IP to a single LIF neuron whose input is a spike (Dirac delta) train randomly generated according to a Poisson process with a mean firing rate of 160 Hz for a duration of 1,000 ms. The details of input generation are described in Legenstein and Maass (2007). The output firing rate is evaluated by the normalized intracellular calcium concentration in (23). Figure 6 compares the output firing distributions generated with no IP and with the three IP rules. Clearly, the proposed rule produces an output distribution close to the desired exponential distribution while without IP tuning the neuron is unable to generate an exponentially distributed output. As shown in Figure 6C, the Voltage Threshold IP rule (Lazar et al., 2007) can only alter the average output firing rate rather than tuning the shape of the output firing rate distribution toward that of an exponential distribution. Figure 6D shows that it is also tricky for the RC IP rule (Li and Li, 2013) to train the neuron to generate an output whose distribution is close to the exponential distribution.

**Figure 7:**
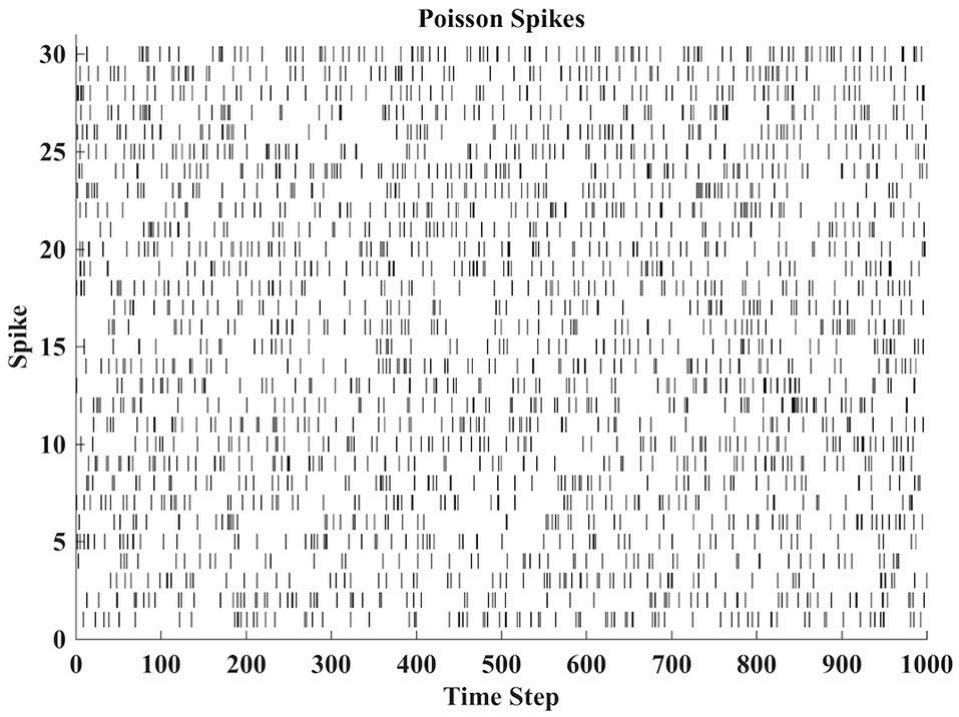
30 Poisson spike trains as input to a fully connected spiking neural network of 100 LIF neurons.

Next, more interestingly, we examine the behavior of IP tuning in a spiking neural network. In this case, we set up a fully connected recurrent network of 100 LIF neurons. There are 30 external inputs with each being a Poisson spike train with a mean rate of 80 Hz and a duration of 1, 000*ms* as shown in Figure 7. Each input is connected to 30 neurons through synaptic whose weights are set to -8 or 8 with equal probability. The synaptic weights between the reservoir neurons in the network are uniformly distributed between -1 and 1. This neural network is similar to the reservoir network used in Schrauwen et al. (2008).

**Figure 8:**
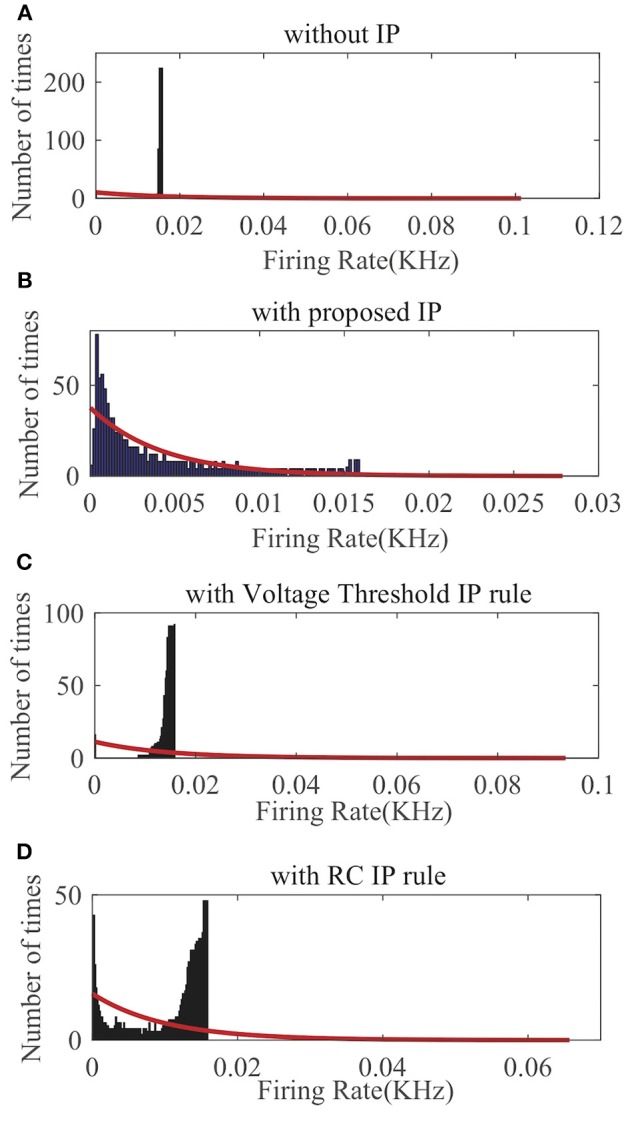
Output firing rate distributions of one spiking neuron in a fully connected network. **(A)** without IP tuning, **(B)** with proposed SpiKL-IP rule, **(C)** with the Voltage Threshold IP rule, and **(D)** with the RC IP rule. The red curve in each plot represents the exponential distribution that best fits the actual output firing rate data.

We randomly choose one neuron and record its output firing rate for a demonstration. As can be seen in Figure 8A, without IP tuning the output distribution is quite different from any exponential distributions. As shown in Figures 8C,D, neither the Voltage Threshold IP rule nor the RC IP rule can produce an output distribution that is reasonably close to an exponential distribution. In contrast, Figure 8B shows that the proposed SpiKL-IP rule leads to excellent results, generating an output distribution that is very close to an exponential distribution. These experiments demonstrate that SpiKL-IP maintains its effectiveness in the more complex network setting where spiking neurons interact with each other while receiving external spike inputs.

### 3.3. Real World Classification Tasks For LSM

Although intrinsic plasticity has been studied for a very long time with many different IP rules proposed, rarely any rule is tested on real-world learning tasks. As a result, it is not clear whether IP tuning is capable of improving the performance for these more meaningful tasks. In this paper, we realize several spiking neural networks based on the bio-inspired Liquid State Machine (LSM) network model and evaluate the performance of IP tuning using realistic speech and image recognition datasets.

**Figure 9:**
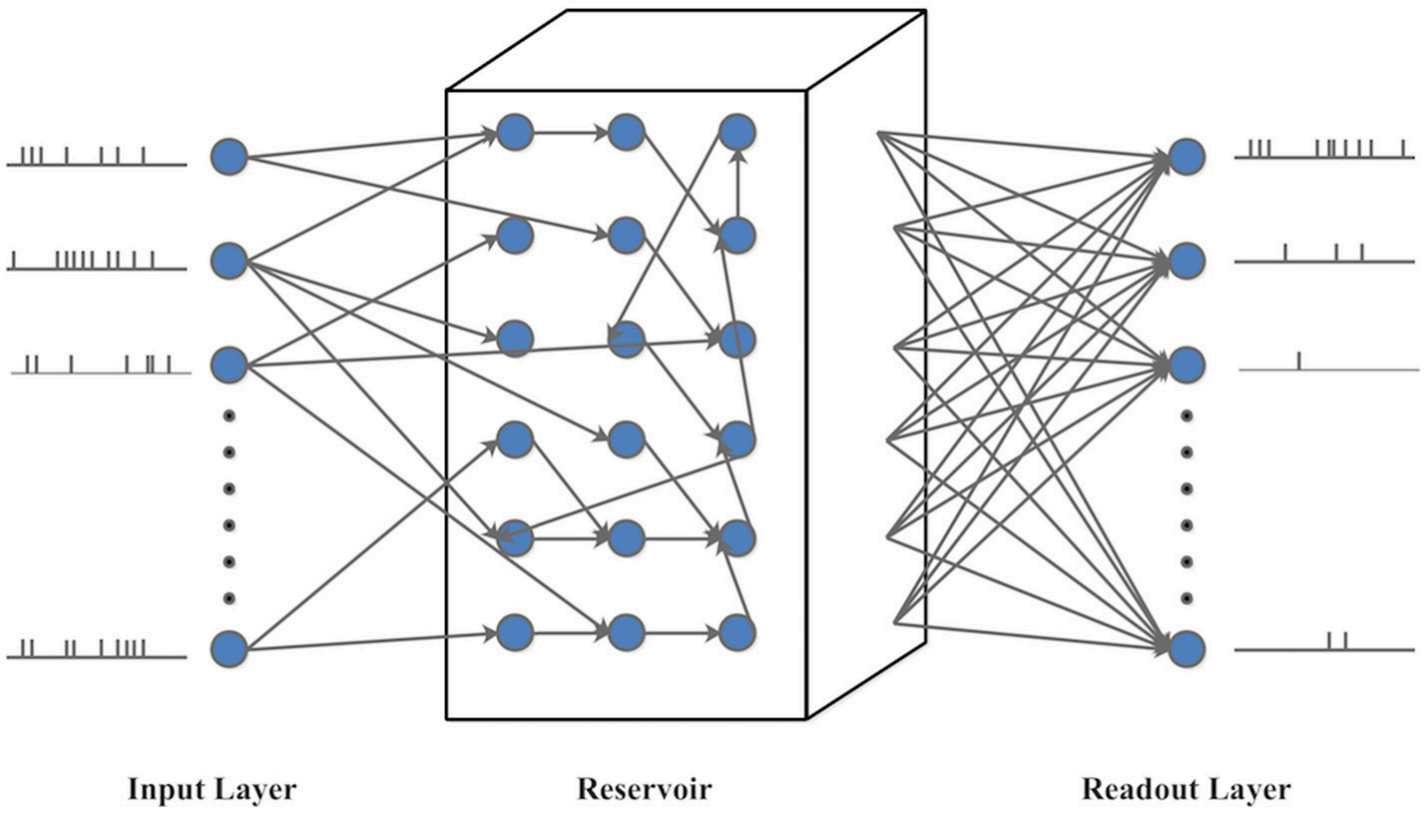
The structure of Liquid State Machine (LSM).

LSM is a biologically plausible spiking neural network model with embedded recurrent connections (Maass et al., 2002). As shown in Figure 9, the LSM has an input layer, a recurrent reservoir, and a readout layer. The reservoir has a recurrent structure with a group of excitatory and inhibitory spiking neurons randomly connected in a way approximating the spatial distribution of biological neurons (Maass et al., 2002). Typically, the synaptic weights between the reservoir neurons are fixed. The input spike trains generate spatiotemporal firing patterns in the reservoir, which are projected onto the readout layer through full connectivity. In this paper, the feedforward plastic synapses between the reservoir neurons and readout are adjusted according to a bio-inspired spike-based online learning rule (Zhang et al., 2015). Several LSMs with different sizes are set up to evaluate the potential impact of an IP rule on classification performance.

For the networks evaluated using TI46, the input layer has 78 neurons. These networks have 135 (3^*^3^*^5), 270 (3^*^3^*^30), 540 (6^*^6^*^15) reservoir neurons, respectively, where each input neuron is randomly connected to 16, 24, 32 reservoir neurons with the weights set to 2 or -2 with equal probability, respectively. Among the reservoir neurons, 80% are excitatory, and 20% are inhibitory. The reservoir is composed of all types of synaptic connections depending on the pre-neuron and post-neuron types including EE, EI, IE, II, where the first letter indicates the type of the pre-synaptic neuron, and the second letter the type of the post-synaptic neuron, and E and I mean excitatory and inhibitory neurons, respectively. The probability of a synaptic connection from neuron a to neuron b in the reservoir is defined as *C* · *e*^−(*D*(*a, b*)/λ)^^2^, where λ is 3, C is 0.3 (EE), 0.2 (EI), 0.4 (IE), 0.1 (II), and D (a, b) is the Euclidean distance between neurons a and b (Maass et al., 2002). The synaptic weights in the reservoir are fixed to 1(EE, EI) or -1(IE, II). For the readout layer, the reservoir neurons are fully connected to 26 readout neurons with the weights randomly generated from -8 to 8 following the Gaussian distribution. All the readout synapses are plastic and trained according to Zhang et al. (2015). When testing an IP rule, it is only applied to the reservoir neurons. The parameters of each neuron are: *V*_*th*_ = 20*mV*, *t*_*r*_ = 2*ms*, μ = 0.2*KHz*, τ_*c*_ = 64*ms*, η_1_ = η_2_ = 5, and α_1_ = α_2_ = 0.1. *R* and τ_*m*_ are initialized to 64Ω and 64*ms*, respectively. The tuning ranges for *R* and τ_*m*_ are again set to [32Ω, 512Ω] and [32*ms*, 512*ms*], respectively. A 5-fold cross-validation scheme is adopted to obtain classification performances. Five hundred epochs are simulated, and the best results are reported.

For the networks evaluated using CityScape, the input layer has 225 neurons. These networks have 27 (3^*^3^*^3), 45 (3^*^3^*^5), 72 (3^*^3^*^8), 135 (3^*^3^*^15) reservoir neurons, each input neuron is randomly connected to 1, 4, 4, 64 reservoir neurons with the weights set to 2 or -2 with equal probability, respectively. Other settings of the networks are the same as those used for the ones evaluated based on TI46.

We also have made our implementation of SpiKL-IP rule for LSM available online^1^.

#### 3.3.1. Speech Recognition Using the TI46 Speech Corpus

The speech recognition task is evaluated on several subsets of the TI46 speech corpus (Liberman et al., 1991). This corpus contains spoken utterances from 16 speakers (eight males and eight females), each speaking 10 utterances of English letters from “A” to “Z”. Before applying to the reservoir, each input sample is first preprocessed by the Lyon ear model (Lyon, 1982), then encoded into 78 spike trains with the BSA algorithm (Schrauwen and Van Campenhout, 2003).

Table 1 demonstrates the classification accuracy for a number of LSMs of different amounts of reservoir neurons with and without the proposed SpiKL-IP rule based on different subsets of the TI46 speech corpus. The 260-samples subset is a single speaker subset while ones with 520, 1,040, 2,080, 3,120, 4,160 samples contain 2, 4, 8, 12, and 16 speakers, respectively. It shall be noted that as the number of speakers increases, the recognition task becomes increasingly challenging. To the best knowledge of the authors, there exists no prior reported success on recognizing multiple-speaker subsets using spiking neural networks. As shown in Table 1, the recognition performs drops rapidly as the number of speakers increases without SpiKL-IP. In comparison, the use of SpiKL-IP can significantly boost the recognition accuracy by up to more than 16%. Moreover, SpiKL-IP leads to higher performance boosts as it is applied to smaller networks or more challenging subsets of greater numbers of speakers and samples.

**Table 1:** The performances of LSM-based speech recognition with and without the proposed SpiKL-IP rule evaluated using the single and multi-speaker subsets of the TI46 Speech Corpus.

**Dataset size**	**Reservoir size**	**Without IP (%)**	**With IP (%)**
260 (1 Speaker)	90	88.46	97.31
	135	92.30	98.46
520 (2 Speakers)	135	86.15	92.31
	270	89.04	95.58
1,040 (4 Speakers)	135	79.04	87.69
	270	84.62	93.37
2,080 (8 Speakers)	270	72.69	86.95
	540	76.59	91.96
3,120 (12 Speakers)	270	72.17	84.25
	540	77.49	90.64
4,160 (16 Speakers)	270	70.76	83.98
	540	76.19	88.58

**Figure 10:**
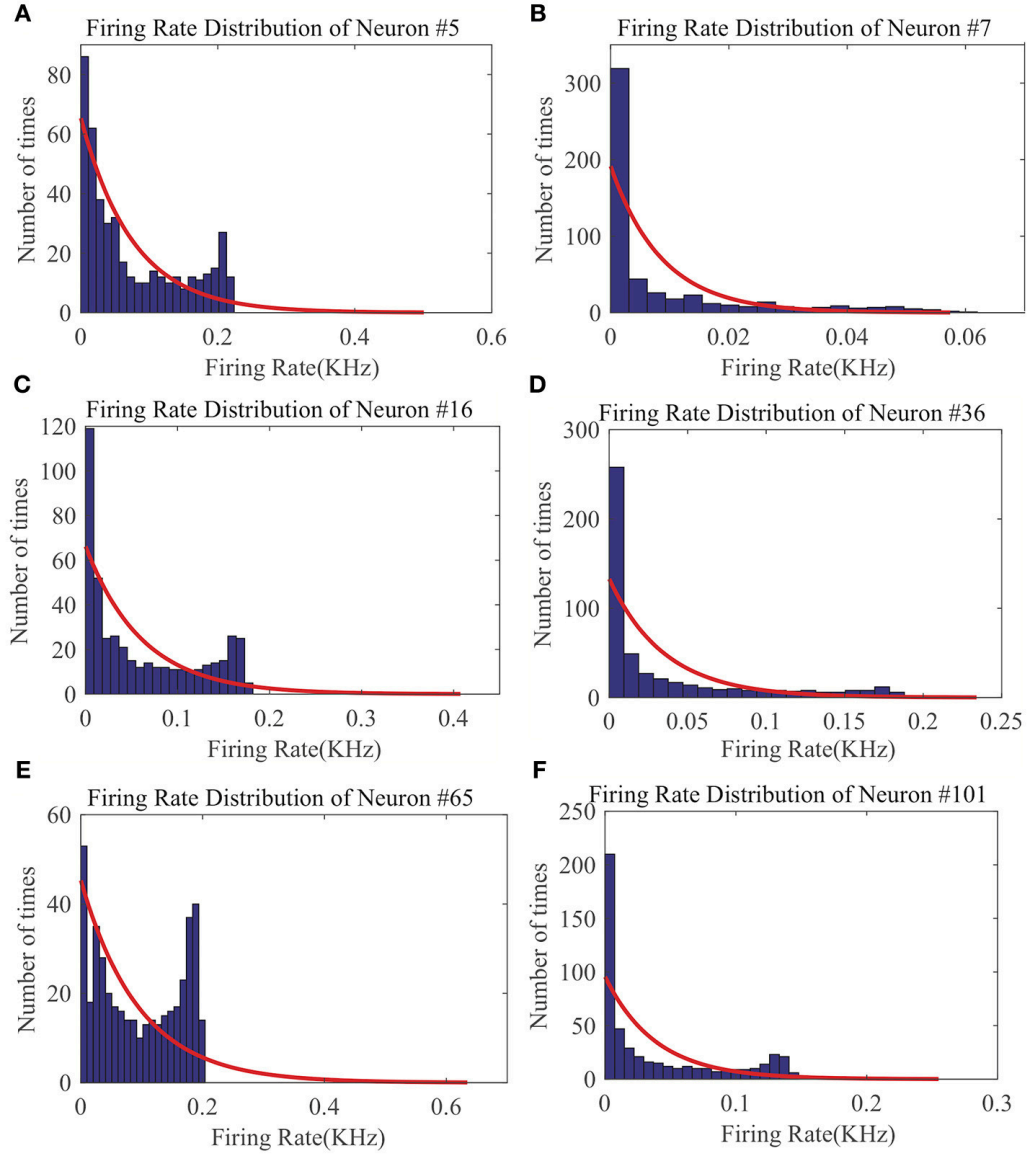
The output firing distributions of six reservoir neurons in an LSM after the reservoir is trained by SpiKL-IP. The red curve in each plot represents the exponential distribution the best fits the actual output firing rate data. **(A)** Firing rate distribution of Neuron #5, **(B)** Firing rate distribution of Neuron #7, **(C)** Firing rate distribution of Neuron #16, **(D)** Firing rate distribution of Neuron #36, **(E)** Firing rate distribution of Neuron #65, and **(F)** Firing rate distribution of Neuron #101.

**Figure 11:**
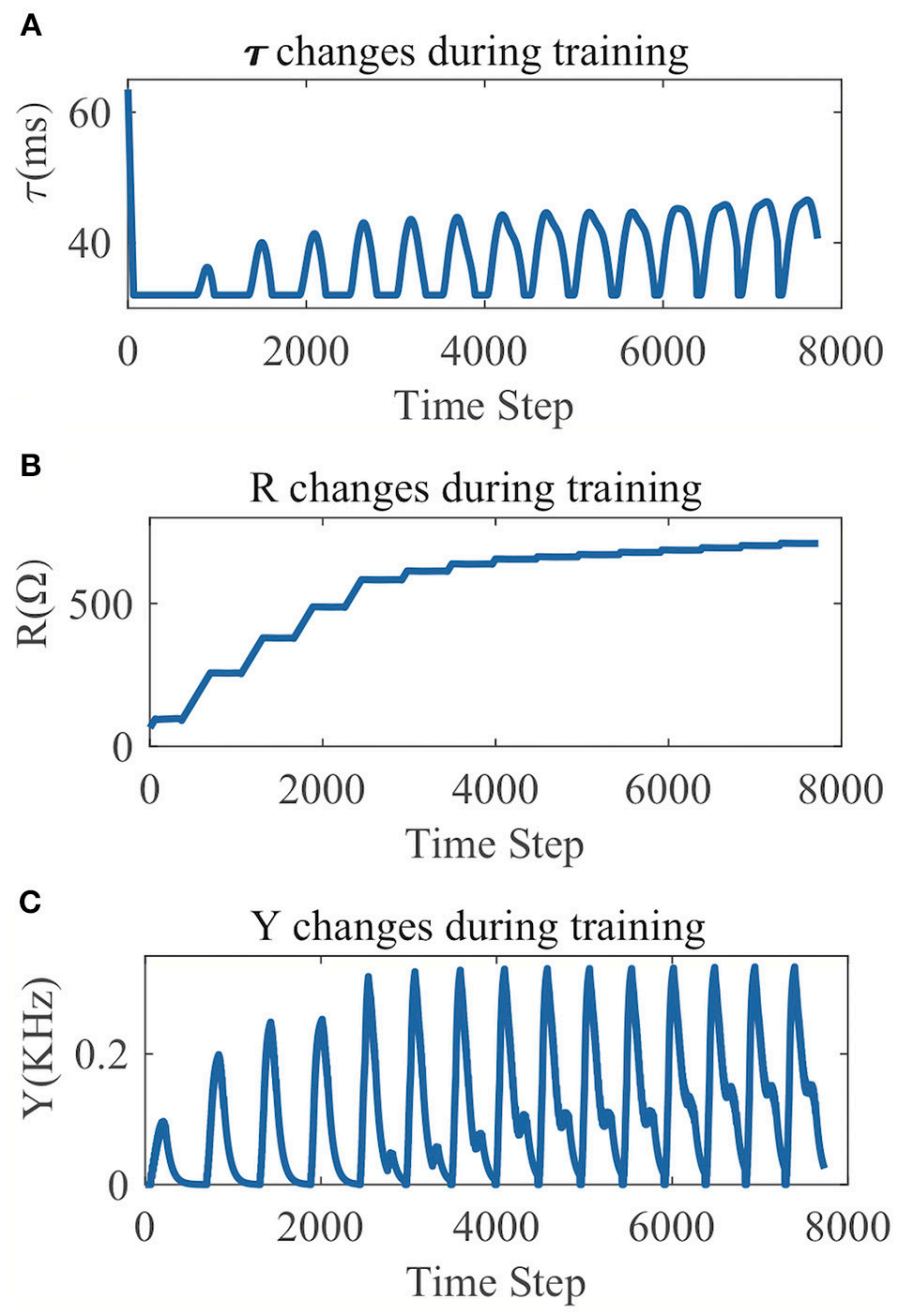
The parameter tuning and firing rate adaption by SpiKL-IP for a reservoir neuron in an LSM during 15 iterations of training over a single speech example. **(A)** Tuning of the membrane time constant τ_*m*_, **(B)** tuning of the leaky resistance *R*, and **(C)** adaptation of the Output firing rate.

From the LSM with 135 reservoir neurons, we randomly choose six neurons and record their firing responses on one of the speech samples after a few initial training iterations. Figure 10 shows that most neurons' responses can follow the exponential distribution, demonstrating that the proposed SpiKL-IP rule can tune neurons to generate outputs with a distribution close to the exponential distribution in a complicated network. Figure 11 shows the learning curves of *R* and τ_*m*_ for a reservoir neuron when one speech sample is repeatedly applied to the network for 15 iterations. Figure 11B shows that the value of *R* monotonically increases over time and finally converges under the proposed IP rule. However, Figure 11A shows that the value of τ_*m*_ fluctuates in every iteration without converging to a fixed value, but its trajectory exhibits a stable periodic pattern toward later iterations. This may be understood by the fact that to produce the desired exponential firing rate distribution, at least one of the two intrinsic neural parameters shall be dynamically adapted in response to the received time-varying input. Figure 11C shows the adaptation of the output firing rate *y*, which has also reached to a stable periodic pattern toward the end of the training process.

**Figure 12:**
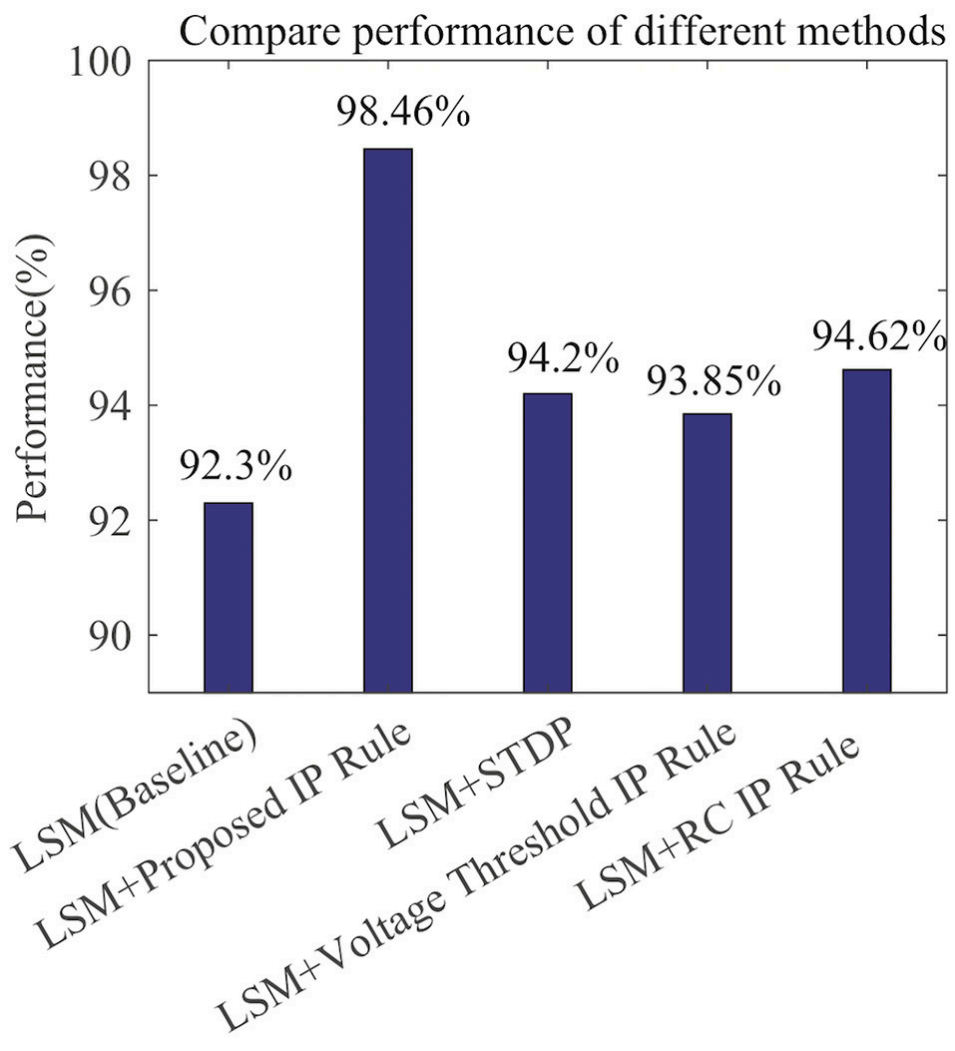
Speech recognition performances of various learning rules when applied to a LSM with 135 reservoir neurons. The performance evaluation is based on the single-speaker subset of the TI46 Speech Corpus. (1) LSM (Baseline): with the settings and supervised readout learning rule in Zhang et al. (2015) and no reservoir tuning. All other compared networks add additional mechanisms to the baseline. (2) LSM+Proposed IP Rule: with additional reservoir neurons tuning using SpiKL-IP. (3) LSM+STDP: with additional reservoir neurons tuning using the STDP rule in Jin and Li (2016); (4) LSM+Voltage Threshold IP Rule: with additional reservoir neurons tuning using the IP rule in Lazar et al. (2007). (5) LSM+RC IP Rule: with additional reservoir neurons tuning using the IP rule in Li and Li (2013).

Figure 12 compares the recognition performances of several LSMs all with 135 reservoir neurons reported in related works. The performances are evaluated based upon the single-speaker subset with 260 samples. We adopt the LSM in Zhang et al. (2015) which makes use of a spike-based supervised learning rule for training the readout synapses and has no IP tuning as a baseline. The LSM in Jin and Li (2016) adds spike-timing-dependent plasticity (STDP) rule to the baseline to train the synaptic weights between reservoir neurons. On top of the baseline, we further implement the Voltage Threshold IP rule (Lazar et al., 2007), the RC IP rule (Li and Li, 2013), or the SpiKL-IP rule to tune the reservoir neurons. The proposed rule produces the highest recognition accuracy improvement of more than 6% over the baseline LSM.

#### 3.3.2. Image Classification Using the CityScape Dataset

The image classification task is based on the CityScape dataset (Cordts et al., 2016) which contains 18 classes of images of semantic urban scenes taken in several European cities. Each image is segmented and remapped into a size of 15 × 15, and then encoded into 225 input Poisson spike trains with the mean firing rate proportional to the corresponding pixel intensity. There are 1, 080 images in total.

Table 2 summarizes the classification accuracy of four LSMs of different sizes with or without the SpiKL-IP rule. For each comparison, an LSM which is set up according to Zhang et al. (2015) and incorporates the same spike-based supervised learning rule of Zhang et al. (2015) for training the readout synapses without IP tuning is used as a baseline. It can be observed that the application of SpiKL-IP leads to noticeable performance improvements. For example, in the case of LSM with 45 reservoir neurons, the performance is improved from 91.74% to 94.44%.

**Table 2:** The performances of LSM-based image classification with and without the proposed SpiKL-IP rule evaluated using the CityScape image dataset.

**Reservoir size**	**Without IP (%)**	**With IP (%)**
135	96.60	97.78
72	94.90	96.48
45	91.74	94.44
27	87.33	90.19

## 4. Discussion

While intrinsic plasticity (IP) was attempted for spiking neurons in the past, the prior IP rules lacked a rigorous treatment in their development, and the efficacy of these rules was not verified using practical learning tasks. This work aims to address the theoretical and practical limitations of the existing works by proposing the SpiKL-IP rule. SpiKL-IP is based upon a rigorous information-theoretic perspective where the target of IP tuning is to produce the maximum entropy in the resulting output firing rate distribution of each spiking neuron. The maximization of output entropy, or information transfer from the input to the output, is realized by producing a targeted optimal exponential distribution of the output firing rate.

More specifically, SpiKL-IP aims to tune the intrinsic parameters of a spiking neuron while minimizing the KL-divergence from the targeted exponential distribution to the actual output firing rate distribution. However, several challenges must be addressed as we work toward achieving the above goal. First, we rigorously relate the output firing rate with the static input current by deriving the firing-rate transfer function (FR-TF). FR-TF provides a basis for allowing the derivation of the SpiKL-IP rule that minimizes the KL-divergence. Furthermore, we cast SpiKL-IP in a suitable form to enable online application of IP tuning. Finally, we address one major challenge associated with applying SpiKL-IP under realistic contexts where the input current to each spiking neuron may be time-varying, which leads to the final IP rule that has no dependency on the instantaneous input level and effectively tuning the neural model parameters based upon averaged firing activities.

In the simulation studies, it is shown that SpiKL-IP can produce excellent performances. Under various settings, the application of SpiKL-IP to individual neurons in isolation or as part of a larger network robustly creates the desired exponential distribution for the output firing rate even when the input current is time varying. The evaluation of the learning performance of SpiKL-IP for real-world classification tasks also confirms the potential of the proposed IP rule. When applied to the reservoir neurons of LSM networks, SpiKL-IP produces significant performance boosts based on the TI46 Speech Corpus (Liberman et al., 1991) and the CityScape image dataset (Cordts et al., 2016).

Our future work will explore the potential of integrating IP tuning with Hebbian unsupervised learning mechanisms, particularly spike-timing-dependent plasticity (STDP). Jin and Li (2017) and this work respectively demonstrate that STDP and IP are effective in tuning recurrent spiking neural networks, i.e., reservoirs, and boosting the overall learning performance. Moreover, it has been suggested by Lazar et al. (2007) and Li et al. (2018) that STDP and IP may be complementary to each other. On the other hand, Watt and Desai (2010) and other related works reveal one limitation of STDP, i.e., the application of STDP can lead to network instability due to the positive feedback mechanisms created. Nevertheless, concerning the potential instability caused by STDP, it may be argued that the joint application of STDP and IP could be beneficial. This is because IP is intrinsically self-stabilizing, which may contribute to the prevention of runaway potentiation caused by STDP. We will also implement the SpiKL-IP rule on noisy leaky-integrate and fire neuron model (Brunel and Sergi, 1998) to evaluate the ability of the SpiKL-IP rule standing against noise. Moreover, since non-Hebbian plasticity and IP are supposed to work together in biological neurons (Watt and Desai, 2010), we can further explore the effects of combining Hebbian unsupervised plasticity, non-Hebbian plasticity, and intrinsic plasticity to maintain the homeostasis of networks.

## Author Contributions

WZ and PL developed the theoretical approach for IP tuning of spiking neurons and the SpiKL-IP rule. WZ implemented SpiKL-IP and related learning rules and performed the simulation studies. WZ and PL wrote the paper.

### Conflict of Interest Statement

The authors declare that the research was conducted in the absence of any commercial or financial relationships that could be construed as a potential conflict of interest.

## Footnotes

^1^https://github.com/stonezwr/SpiKL-IP
